# Development of a Smart Leg Splint by Using New Sensor Technologies and New Therapy Possibilities

**DOI:** 10.3390/s21155252

**Published:** 2021-08-03

**Authors:** José María De Agustín Del Burgo, Fernando Blaya Haro, Roberto D’Amato, Alonso Blaya, Juan Antonio Juanes Méndez

**Affiliations:** 1 Campus Miguel de Unamuno, Universidad de Salamanca, 37007 Salamanca, Spain; id00792219@usal.es (J.M.D.A.D.B.); jajm@usal.es (J.A.J.M.); 2 ETSIDI-Departamento de Ingeniería Mecánica, Química y Diseño Industrial, Universidad Politécnica de Madrid (UPM), Ronda de Valencia 3, 28012 Madrid, Spain; fernando.blaya@upm.es; 3 Departamento de Fisioterapía de la, Universidad Europea de Madrid, 28670 Madrid, Spain; alonso.blaya.sp@gmail.com

**Keywords:** smart splint, rehabilitation therapy, IoT, AM technique, customized medicine, health monitoring

## Abstract

Nowadays, after suffering a fracture in an upper or lower limb, a plaster cast is placed on the affected limb. It is a very old and efficient technique for recovery from an injury that has not had significant changes since its origin. This project aims to develop a new low-cost smart 3D printed splint concept by using new sensing techniques. Two rapidly evolving Advanced Manufacturing (AM) technologies will be used: 3D scanning and 3D printing, thus combining engineering, medicine and materials evolution. The splint will include new small and lightweight sensors to detect any problem during the treatment process. Previous studies have already incorporated this kind of sensor for medical purposes. However, in this study it is implemented with a new concept: the possibility of applying treatments during the immobilization process and obtaining information from the sensors to modify the treatment. Due to this, rehabilitation treatments like infrared, ultrasounds or electroshock may be applied during the treatment, and the sensors (as it is showed in the study) will be able to detect changes during the rehabilitation process. Data of the pressure, temperature, humidity and colour of the skin will be collected in real time and sent to a mobile device so that they can be consulted remotely by a specialist. Moreover, it would be possible to include these data into the Internet of Things movement. This way, all the collected data might be compared and studied in order to find the best treatment for each kind of injury. It will be necessary to use a biocompatible material, submersible and suitable for contact with skin. These materials make it necessary to control the conditions in which the splint is produced, to assure that the properties are maintained. This development, makes it possible to design a new methodology that will help to provide faster and easier treatment.

## 1. Introduction

Currently, 3D technology with biocompatible materials is being used for many applications [1] such as in dental reconstruction, by using the Selective laser fusion technique (SLM) [2]; or the manufacture of orthopedic prostheses [3,4] by using Fusion Deposition Modelling (FDM). In both cases, the process consists of scanning the patient’s teeth or limb and after the corresponding processing, it is possible to manufacture a custom piece [5]. Moreover, in recent years, many studies have shown that 3D printing techniques make it possible to engineer anatomical parts of the human body such as tissues and organs [6,7,8].

The advantages offered by 3D technology applied to health science are of fundamental importance for the patient’s recovery, especially for people with injuries whose only therapy is the immobilization of a part of the body. Furthermore, the digitalization and 3D prototyping of medical artifacts for immobilization, allows during the design phase, to create a solid product with non-continuous surfaces, while maintaining rigidity and resistance [5]. It also ensures a level of hygiene that cannot be attained with conventional immobilization techniques, thanks to the use of bio-plastic materials compatible with humid environments [9,10].

In addition, there is another great advantage of using new splints instead of tradition-al ones. These new splints allow the design of dedicated therapeutic windows (material-free areas) to apply treatments that must be applied directly to the skin, and therefore, cannot be applied otherwise. A simple window will allow cures for the case of wounds, dermatological pathologies or surgeries. The generation of windows on the splint, also allows for periodic visual medical inspection during the immobilization phase and rapid decisions on the evolution of the lesions.

This project focuses on the development and prototyping of an immobilization smart splint for a leg using a 3D scanner, processed from the obtained file, and its subsequent printing with biocompatible material. Taking advantage of new manufacturing techniques and novel technologies in sensors and electronics, it is proposed to bring the reinterpretation of this technique from the 10th century to our present time, by using new sensing and electronic technologies [11,12], and the use of the Internet of Things [13,14], applied in medical applications. It will be possible to develop intelligent splints designed according to the lesion and morphology of the patient.

The splint will be designed exclusively for each individual, and it may be possible to have splints prepared prior to injury, especially in high-level athletes. This would make it possible to modify the rehabilitation treatments with the data acquired by the sensors during the process [15]. In this study, the steps to manufacture a smart splint with AM technology starting from the digitalization of the leg to immobilization will be presented.

The sensing technologies will be presented for data acquisition in the IoT-based healthcare system. Based on the previous study [16] the parameters that will be monitored are temperature, humidity, pressure and skin colour. The combination of these, can indicate different problems that the patients may be suffering [17,18], such as inflammation. In order to obtain the prototype of the splint, the windows for the accommodation of the rehabilitation therapies and the type of treatment will be considered. In fact, the application of treatments in the immobilization phase has a substantial influence on the evolution of the injury and on mobility rehabilitation of the limb. Moreover, the splint will allow visual access through windows and direct contact with the skin.

There are different considered treatments for the case under study. Lymph drainage uses Combined Decongestive Physiotherapy (CDP) for lymphedema which is recognized and reimbursed by insurance companies [19]. The basis of lymph drainage is to create different pathways through which lymph can flow. Electrodes may be used for this purpose [20,21]. Iontophoresis is one of the most used during rehabilitation treatment for delivery of anti-inflammatory and painkiller medication in the parts of the human body affected by inflammatory processes of the musculoskeletal system [22,23,24]. This way, some medications are able to pass through the skin and produce their effect, circumventing the digestive tract and without the need to have them administered by injection [25,26,27]. The Therapeutic Ultrasound technique is used in rehabilitation therapies for its mechanical, analgesic and circulatory effects and it should be applied in an aqueous medium (gel or immersion), something impossible with traditional splints [28]. It also improves the elasticity of the tissues [29]. It reduces the symptoms of inflammation and promotes tissue regeneration [30,31]. Normally the bones and the tendon connection area have low absorption capacity, so the frequency will depend on the injury [32,33]. Laser therapy accelerates energy metabolism and tissue synthesis as reported by several studies, [34,35,36]. Low-level laser stimulates the cells and modulates inflammatory processes [37,38,39], produces an increment of collagen production in the tendons structure [30,40,41,42] and reduces the levels of inflammation [43,44]. Electrostimulation for neuromuscular-skeletal mild to moderate pain is based on transcutaneous electrical nerve stimulation (TENS) and percutaneous electrical nerve stimulation (PENS) [45]. There are also different studies about the effects of electrostimulation on muscular strength and functional capacity in patients with osteoarthritis of the knee [46].

These techniques are only some examples considered in this study, that may for instance be applied to injuries or lesions in arms [47], knees [48], elbows [49] or Achilles tendons [50]. However, by using 3D splints, it could also be possible to apply other therapies, considered by a specialist [51].

As part of the process, objectives that will be considered are:

- Development of the protocol for scanning, meshing, designing and producing the splint.
- Development of mobile application for sensor monitoring.
- Implementation of an algorithm to detect inflammation through changes in pressure, temperature or colour of the affected area.
- Study on the measurement of humidity on the internal face of the splint, according to the discussion of convenience that will be done later in the article.

The full process will include obtaining the 3D scanning model using a 3Dscanner first, as well as the verification of the data obtained. After this, the file provided by the scanner will be modified using CAD Software to obtain a final model. This model will incorporate the sensors windows to apply the different treatments. Finally, the model will be produced and the software and electronics will be integrated.

## 2. Materials and Methods

The steps to model, design, manufacture and assemble the 3D smart and functional leg splint are presented and detailed below. The leg of a 29-year-old healthy volunteer was used as a model. The same proposed methodology could be applied to other parts of the body [10]. In fact, it is analogous to others lesions that may require immobilization of the upper limb articulations, such as the wrist [16].

Furthermore, the different rehabilitation treatments and sensors are detailed, to be implemented on the design of the splint.

### 2.1. Sensing Technologies

For this study, the parameters that will be monitored are temperature, humidity, pressure and skin color. The information recorded by these sensors is combined to determine if there is any kind of unexpected evolution of the treatment. In total, the implemented sensors are: two temperature sensors DS18B20 [52] in contact with the skin, one temperature and humidity sensor between the skin and the splint (DHT22) [53], two pressure sensors DF9-40 [54] placed in two different axes of the splint (X, Y), and one color sensor TCS34725 [55]. The shocks and vibrations that may be produced by the human body are not critical to the performance and durability of these sensors. Moreover, when an injury of these characteristics is being treated, it is due to the requirement of immobilization. Therefore, the shock and vibrations are controlled. In addition, some of these sensors have been tested in a previous study, where it is detailed that accuracy of the selected sensors is enough for the purpose [16]. Details of these sensors are shown in Table 1.

The electronic design and configuration of the temperature, humidity and pressure sensors are explained in a previous study [16]. However, the color sensor has been incorporated for the first time in this study.

During the healing process, a bruise will usually go through different colors. It often starts red because fresh, oxygen-rich blood has newly pooled underneath the skin. After around 1–2 days, the blood begins to lose oxygen and change color. A bruise that is a few days old will often appear blue, purple or even black. In about 5–10 days, it turns a yellow or green color. These colors come from compounds called biliverdin and bilirubin that the body produces when it breaks down hemoglobin. After 10–14 days, it will turn to a shade of yellowish-brown or light brown [56,57].

**Figure 1:**
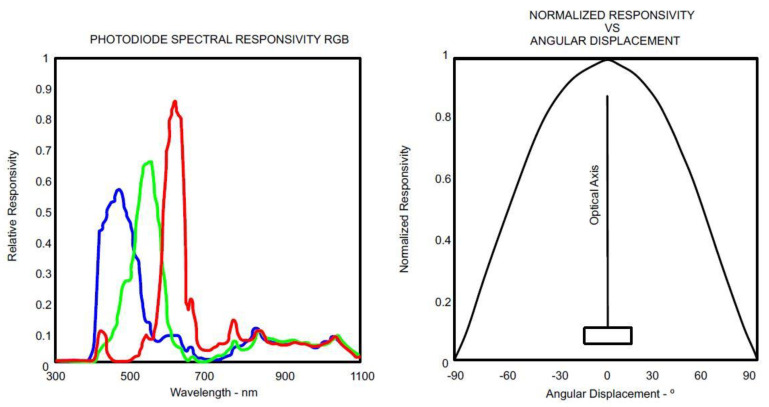
TCS3472 sensor responsivity graphs (**a**,**b**) [55].

The TCS3472 sensor provides a digital return value of three components of the light (red, green and blue), and it also clears the light detection values. It includes an IR blocking filter, which minimizes the IR spectral component of the light, allowing for accurate color measurements over the skin, where there is not external light. Figure 1 shows graphs of the specific response from this sensor to the light. As Figure 1a shows, the sensor is able to detect different wavelengths by using a spectral photodiode. In this way, it comprises the amount of light of each wavelength that bounces off a surface. Figure 1b illustrates that the highest responsivity of the sensor is in the perpendicular direction of the photodiode surface. This must be considered when the housing of the sensor is designed. This sensor is an ideal solution for varying lighting conditions and materials, due to its sensitivity, the wide dynamic range and the IR blocking filter. The data are transferred via an I2C bus, using the SDA and SCL contacts.

Previous studies show the suitability of different RGB channels and their differences for detection of hematomas or ecchymosis [58,59]. The color sensor detects changing colors of the skin, during the initial inflammation stage, due to ecchymosis [60], which occurs around approximately the first six days [61,62]. The main purpose of using an RGB sensor, is to detect the variation in red, green and blue components of the light. The device incorporates an emitter of white light that bounces off the skin and goes back to the receiver. The sensor reads the variation of the three components, in comparison to the original white light. This way it is possible to know what component is absorbed, and therefore, the color of the skin. It is not necessary to modify the gain value or other parameters, as the sensor is already configured. Moreover, as the initial stage of the lesion that must be monitored has a duration of one week, the durability is not a critical parameter. The sensor reads the values in terms of RGB (red, green and blue) between 0 and 255.

### 2.2. Considered Therapies during the Design Process

**Figure 2:**
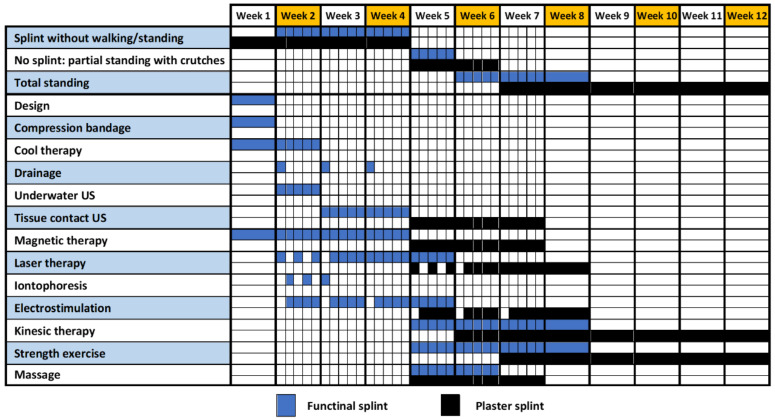
Chronogram of rehabilitation with new techniques applied [10].

According to the previous study [10], the treatment programming is shown in black color in Figure 2. Traditional splints do not allow the application of physiotherapy techniques until the device is removed. However, by using new smart splints, it is possible to apply the treatments as soon as the injury occurs. It is possible to see the new timing possibilities in blue color in Figure 2.

This reduction involves at least 30% of the treatment period. Moreover, the application of physiotherapy techniques during the immobilization phase contributes to the prevention of joint, muscular and vascular complications, derived from the application of retention devices in the initial phase of the treatment.

### 2.3. 3D Model and Design

The first step for the design of the leg splint is the full scan of the injured limb in order to achieve the shape that perfectly suits the patient. In this case, the 3D Systems Sense^®^ scanner is used to perform different sweeps of the leg. The leg to be immobilized must be kept suspended during this process so that a detailed scan of the bottom surface can also be made. The digitalization of this part is a fast step, but it is vital to getting a correct model later, so it is critical to the rest of the process. Table 2 shows the technical specifications of the scanner.

**Figure 3:**
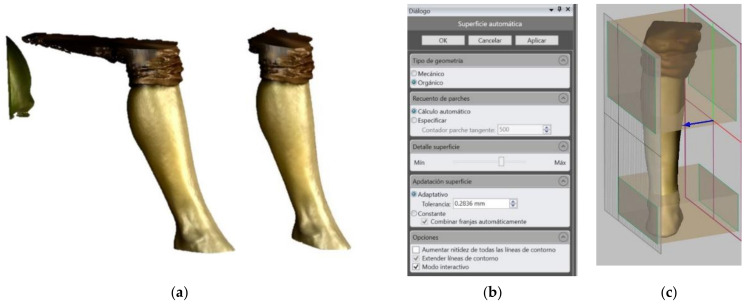
(**a**) Original and treated points cloud in Geomagic Software; (**b**) Mesh surface creation parameters; (**c**) Mesh manipulation.

After this process, a points cloud is obtained and sent to the 3D CAD Geomagic Freeform Software (3D Systems, Inc., Rock Hill, SC, USA). This program, designed to manipulate no-geometric models, eliminates outlier data points from the points cloud [63,64]. These points are normally created during the scanning process due to disturbances from the light. After this, the software transforms the points cloud into a mesh surface. This surface will be the basis for the creation of the solid that will constitute the final splint (Figure 3a). Once the mesh is created, it is possible to manipulate it, creating offsets or cuts. It is necessary to create an offset of 0.5mm from the obtained surface. This way there will be slight free space between the limb and the splint. It is also at this point when the area of the limb that will be covered with the splint is decided (Figure 3b,c). Finally, a new mesh is generated with an offset of 3.7mm, which will be the thickness of the splint. That thickness will allow the different sensors to be fixed and a splint ruggedized enough to be built, based on previous studies about splints produced by fused deposition modeling and PLA mechanical properties [16,65].

**Figure 4:**
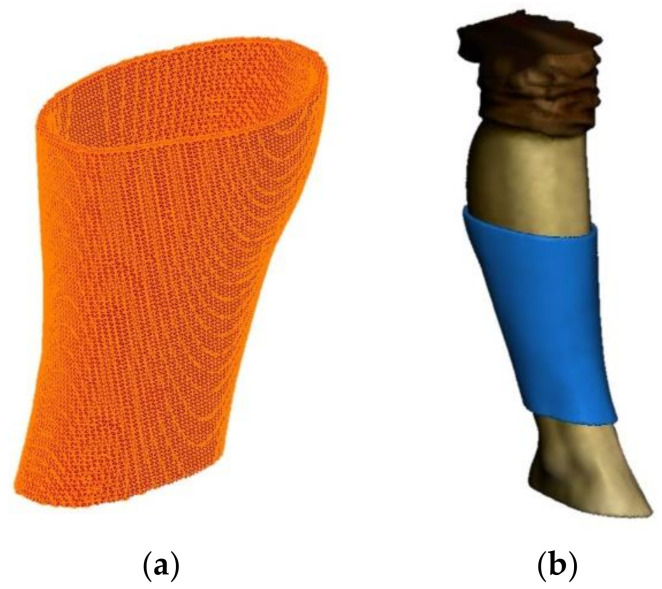
Mesh of the splint (**a**) and mesh over the original 3D model (**b**).

**Figure 5:**
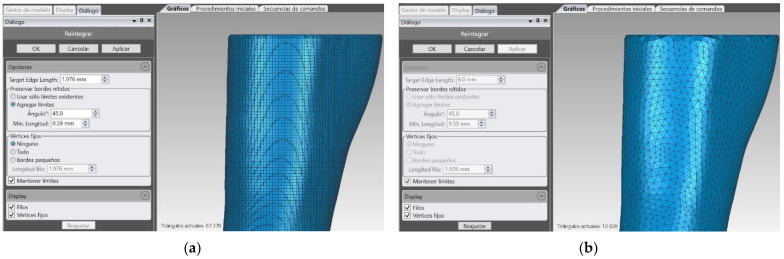
Original mesh (**a**) and reduced mesh (**b**).

It is possible to see the generated mesh of the splint and the splint over the original model in Figure 4. However, at this point, it is just a mesh. To be able to easily design the modifications for the treatments and sensors, it is necessary to create a solid body from this mesh surface. This way, it will be possible to import the digital model to a parametric CAD program to manipulate it correctly. This goal is not trivial, as the methodology of parametric modeling and non-parametric modelling programs (organic modelling programs) are complete opposites. Parametric modeling is an approach to 3D CAD in which you capture design intent using features and constraints, which allows users to automate repetitive changes, such as those found in families of product parts. These capabilities, are a great fit for design tasks that involve precise requirements and manufacturing criteria. For example, this is normally used when making families of products that include slight variations of a core design. This supports designs that will need to be modified or iterated on a regular basis and creates models with individual features, such as holes and chamfers, that can be modified or changed. First, the mesh is reduced to be able to manipulate it easily. It is important to reduce the number of triangles, but also to keep the original dimensions and forms. Then, the mesh is sent to 3D CAD Geomagic Design X™ Software (3D Systems, Inc., Rock Hill, SC, USA). With this program it is possible to complete the mesh and to reduce the number of surfaces. Figure 5 shows a total of 67,376 triangles, and after applying the tool, these triangles are reduced to 10,820. This 84% reduction of the mesh maintains the original design with a much smaller file size.

**Figure 6:**
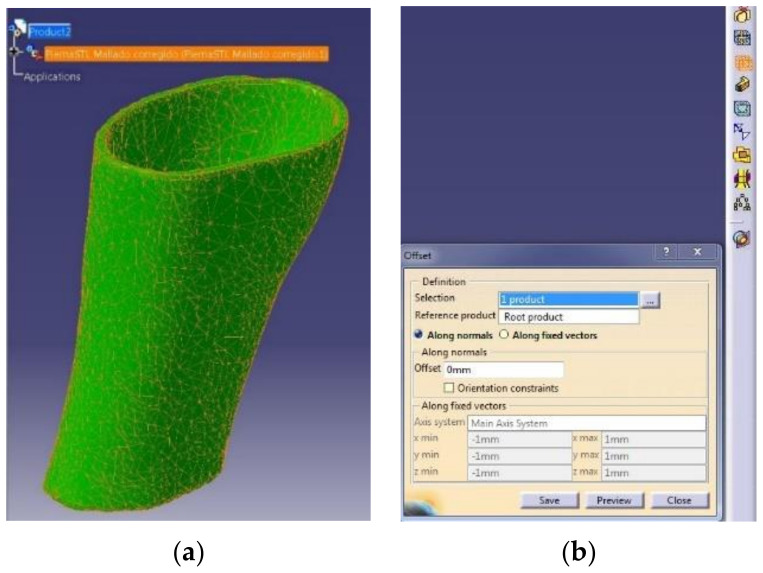
Catia Software. Reduced mesh (**a**) and offset tool (**b**).

There are different steps to be followed using CATIA™ 3DEXPERIENCE^®^ 3D Software (Dassault Systèmes^®^, France). The reduced mesh is imported by using the Assembly Design module. After that, the mesh is converted to an old extension “model” file using the DMU Optimizer module with the tool of applying a 0 offset to the mesh (Figure 6). The generated file is opened and the tree of the design must be copied and pasted to a new part in the Generative Shape Design module. Using this module, it is possible to use the “Joint” tool to create a unique and complex mesh. Finally, this mesh is opened using the Part Design module and the tool “Close Surface” is applied to create the solid body of the splint. After this procedure, the original mesh is converted to a solid body.

**Figure 7:**
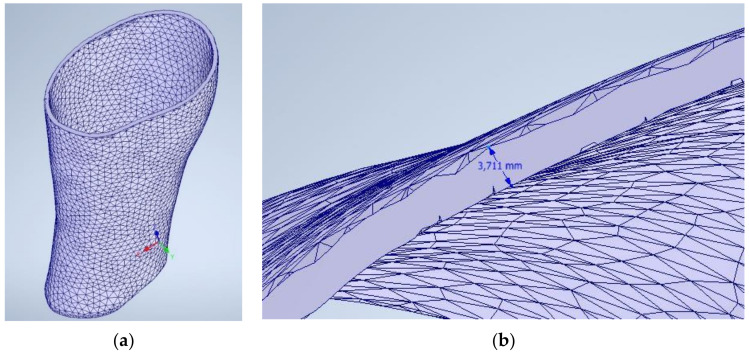
Splint imported from Inventor Autodesk Software (**a**) and dimensional check (**b**).

Thanks to this process, now it is possible to open the file with a parametric modeling software. In this case, Inventor Professional 2020 (Autodesk, Inc, Mill Valley, CA, USA) will be used. If the thickness is correct, this indicates the process has not modified the dimensions in comparison to the original offset that was set (Figure 7), so the process is valid and has not modified the original morphology and dimensions.

Once the model is included in this parametric design software, the procedure to modify the splint accordingly to the necessities begins.

**Figure 8:**
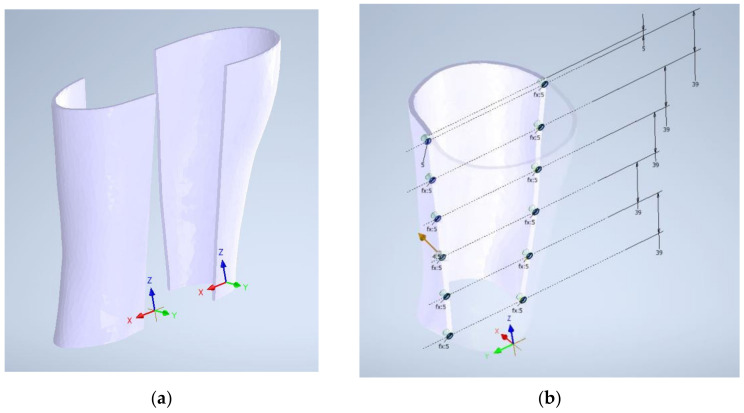
Division of the splint (**a**) and magnet housing design (**b**).

The splint is divided in two parts so that the patient can correctly fix the real model later, joining these parts with a mechanical closure. In this case, twelve magnets of 3mm diameter and 3 mm high are set in each part. However, other fixing systems could be used. Figure 8 shows this process.

**Figure 9:**
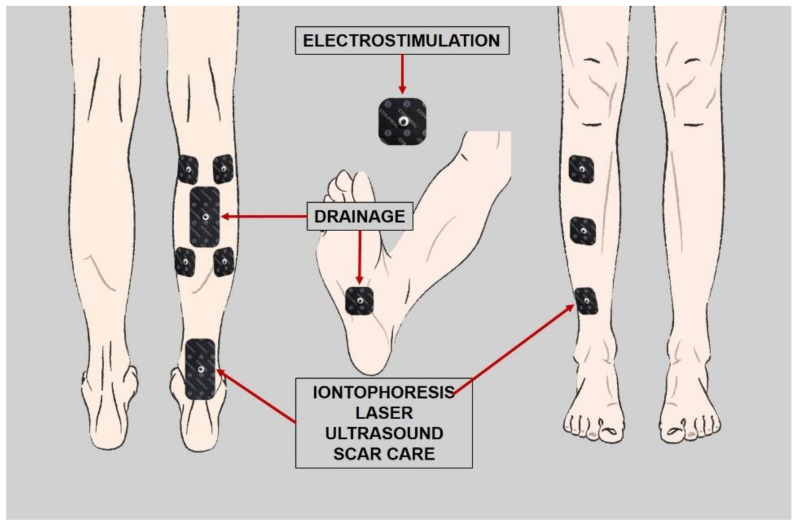
Therapeutic windows to be made in the splint.

At this point, it is necessary to introduce in the splint the different housings for the sensors as well as the windows for the different treatments proposed in Section 2.2. Figure 9 shows the areas where the rehabilitation therapy treatments will be applied, according to the morphology of the limb to be immobilized and previous studies [25,30].

**Figure 10:**
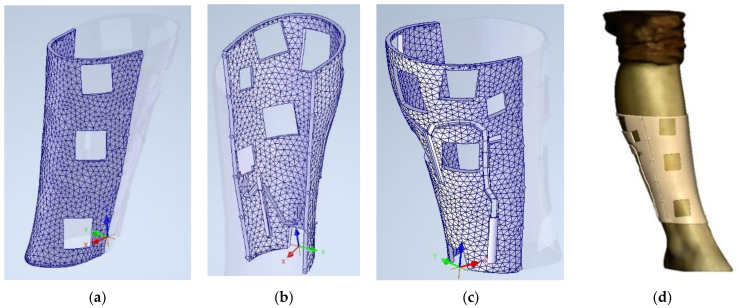
View of the splint: front part (**a**), inside (**b**) outside (**c**) from the back part of the splint. (**d**) designed splint over the scanned model.

Considering these factors, different windows are designed. For electrostimulation, 30 × 30 mm windows are made, whereas for drainage, iontophoresis, laser and ultrasound, the windows have a size of 30 × 40 mm. Furthermore, different housings for the sensors and wiring are considered. Figure 10a–c show both the back and front part after the designing process. Finally, the designed model is opened over the original mesh of the leg, obtained at the beginning of this process, to confirm it fits perfectly (Figure 10d).

### 2.4. Additive Manufacturing

**Figure 11:**
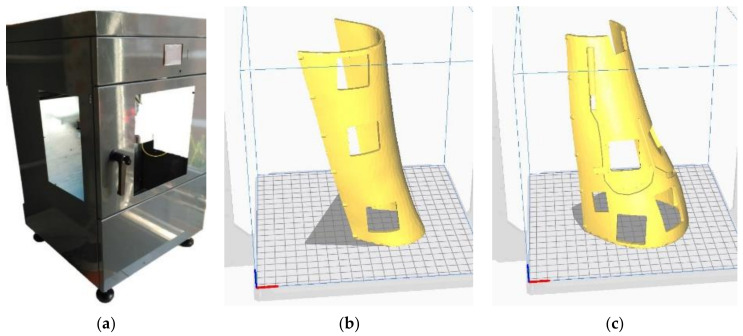
Total-Printer machine (**a**) and the two parts of the splint ready for printing (**b**,**c**).

After the 3D scanning and design process, a *.stl* file is obtained that can be processed by a slicer software that prepares the model to be produced by using AM processing (Figure 11). In this case, a FDM 3D printer “TotalPrinter” machine was used. This printer was designed, developed, and manufactured in the Aditive Manufacturing and Rapid Prototype Laboratory of the Escuela Tecnica Superior de Ingenieria y Diseño Industrial at Technical University of Madrid (Spain). This printer allows controlled and continuous monitoring of printing parameters such as the thickness of the extruded thermoplastic filament [66], the humidity and temperature of the printing chamber and the temperature of the extruder [67] in order to optimize the printing process and to obtain a homogeneous model in terms of adhesion between the printed layers and mechanical characteristics [68].

For producing the splint, PLA filament (see Table 3 for mechanical properties) with a diameter of 1.75 mm was chosen. The material and process should be followed carefully in order to avoid biocompatibility issues [69,70,71]. The vertical position for printing was chosen as shown in Figure 11, taking into account the volume chamber of this machine (200 mm × 200 mm × 400 mm). In Table 4, it is possible to see the printing parameters set in the manufacturing process, following the material manufacturer’s recommendations to obtain a quality and structure rigid enough.

### 2.5. Sensing Technologies

In order to correctly detect the data from the sensors, it is necessary to develop an electronic board for connecting everything easily. It is important to focus on a durable design, with low weight and dimensions. By focusing on these main purposes, a board that will be installed directly to the TTGO OLED Display and Battery Board is designed, shown in Point 2.2 of this study. This board has two analog-to-digital converters (ADC1 and ADC2). The resolution of these converters is 12 bits. A 12 bits ADC means that is able to read $2^{12}$ values between 0 and 5 V. This is 4096 values, so it detects changes of 1.22mv, which is enough for what is required by the selected sensors. ADC1 is used for the sensors, whilst ADC2 is used for the communication between the microprocessor and the Wi-Fi module, which is used to create the web server [72].

**Figure 12:**
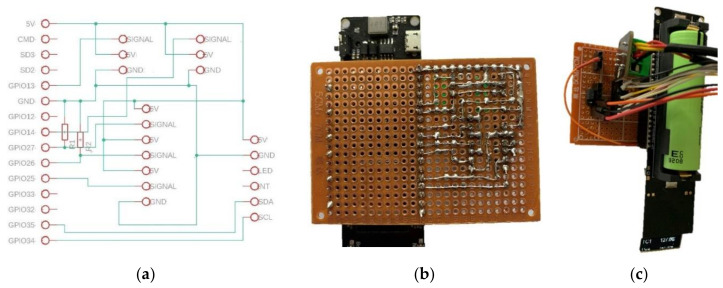
Schematic design of the electronic board for the sensors (**a**), soldering process (**b**) and electronic assembly (**c**).

It is necessary to install different contacts and resistors to properly connect to each sensor. It is possible to see the design and assembly in Figure 12a,b. The final electronic assembly is showed in Figure 12c.

The chosen board, as explained before, can be accessed from other devices through different ways and protocols. It has an OLED display that may show the acquired data (pressures, temperatures, humidity and color change detection). Furthermore, it is possible to create a Bluetooth connection with a mobile phone or similar device. However, in this case, the option of creating a web server is selected. This way, the information may be consulted from any device connected to the internet, anywhere in the world. The great benefit of this, is that a doctor can see the data and follow the treatment in real time from their place of work.

The total real consumption of the electronic board and sensing system is 84.66 mA. Due to the battery has a capacity of 12,210 mAh, the autonomy of the system is around 144 h. This means that the system is able to collect data during six days without charging the battery, what is very significant because the first week after occurring the lesion is the most important period to be analyzed [56,57]. After that, it is possible to replace or charge the battery.

## 3. Results

This section presents the different results obtained after the 3D printing process of the designed model. In addition, the electronic devices are assembled and the sensors are placed in their housings and connected to the electronic board. Finally, the configuration of the ESP32 microprocessor is presented as well as the acquisition of data and results.

**Figure 13:**
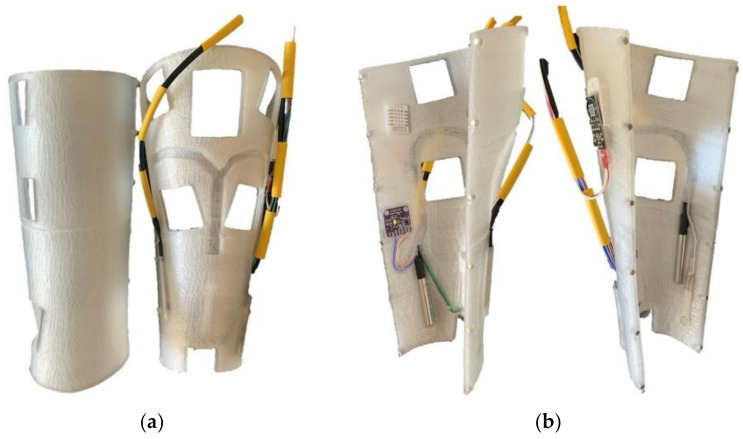
Real splint assembled with the sensors (**a**) and interior view (**b**).

Figure 13a shows the splint prototype obtained with the wiring and sensors fully assembled. In Figure 13b it is possible to see the interior of the splint with the housing for the sensors.

**Figure 14:**
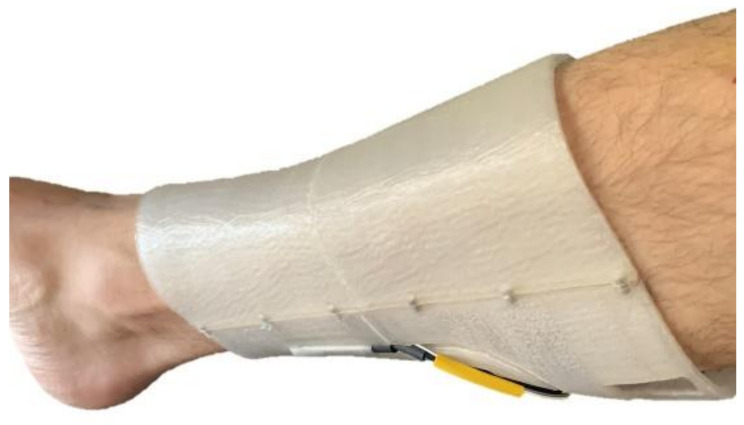
Real model over the leg.

To test the fully assembled leg splint on a real leg, the splint was fixed to a healthy volunteer for 1.5 h (Figure 14). The volunteer was placed in seated position for the tests. However, this new “smart splint” is capable of obtaining data in a continuous way, so the patient does not need to adopt particular positions during the data acquisition. The main purpose of this test, was to collect data so that it could be analyzed if the sensors were able to acquire information correctly. The data collected by the sensors were treated with ESP32 micro and sent to a computer every 3 s by using the serial monitor of the Arduino platform. In the following sections the acquired data will be explained.

### 3.1. Temperatures and Humidity

**Figure 15:**
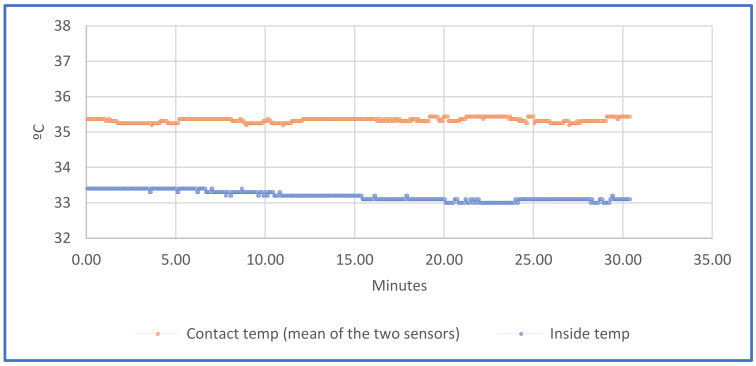
Temperature’s graph.

Two temperature sensors in contact with the skin were placed in two different zones. Moreover, a third sensor not in direct contact with the skin, read the temperature and humidity. The mean temperature of the first two sensors is shown in the color orange, whereas the internal temperature is shown in the color blue (Figure 15). The graph shows that the registered temperature by the sensor is slightly lower than 36 °C. This occurs because the exterior area of the sensor surface is not in real contact with the skin, and this produces a gradient of temperature between the environment and the body. However, what the authors are looking for in this study, is to detect an increment or decrement of the temperature of the located area of the skin, compared with the historical. It is perfectly possible to detect an increase or decrease of the registered temperature, in comparison with the historical data.

**Figure 16:**
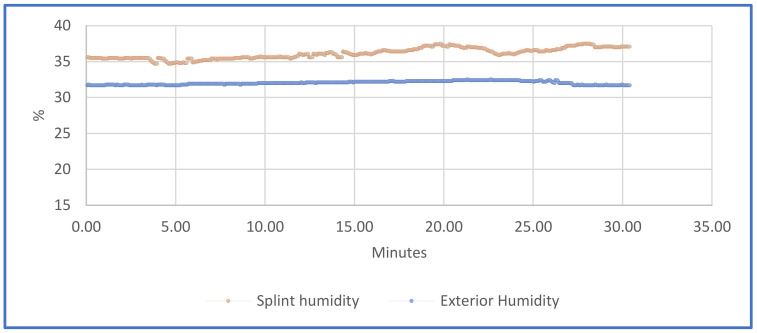
Humidity graph.

Monitoring the humidity inside the splint and its fluctuations allows us to know if the injured area is in a healthy environment and unsuitable for the proliferation of bacteria, especially in cases of surgery or injury. Furthermore, some physiological events that can occur following a trauma have been shown to include changes in skin color due to edema, changes in temperature and an increase in sweating in the area affected by the lesion [73]. In fact, a change in the sudomotor function of the injured part implies central disturbances of thermoregulation [17,74]. Figure 16 shows the percentage of humidity that was registered during the test inside the splint (in orange color) and outside the splint (in blue color).

### 3.2. Pressure

**Figure 17:**
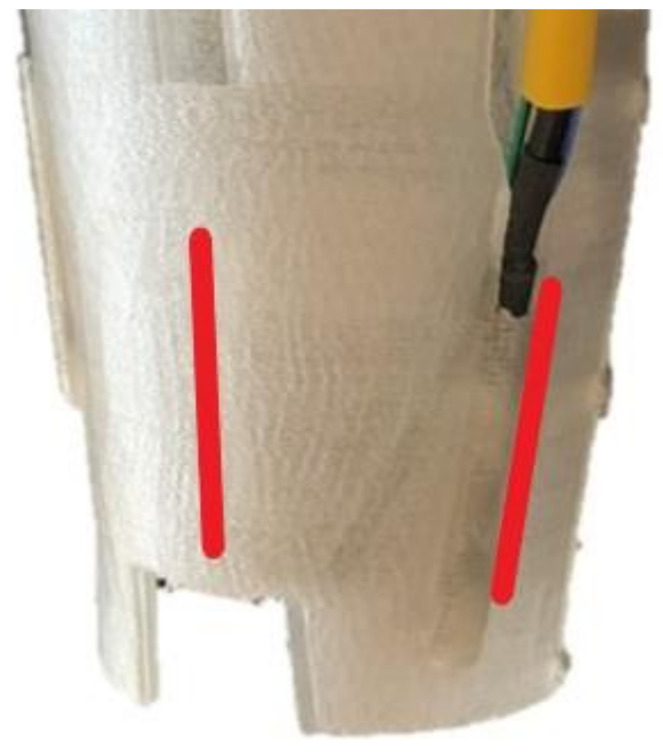
Pressure sensors on the “X” and “Y” axis.

Two sensors for reading pressure are placed. One is placed on the “X” axis and the second on the “Y” axis (Figure 17). The sensors have been placed on these positions for the tests, and the data are collected with the leg in vertical position and weightlessly. However, the potential of these kinds of 3D techniques makes it possible for other medical requirements to also be considered and included during the design process, according to specialist dictated requirements.

**Figure 18:**
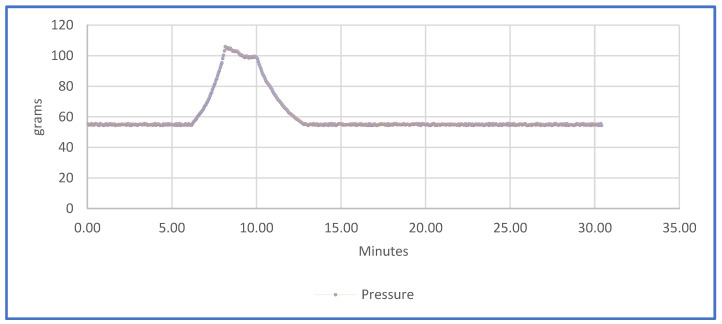
Pressure graph.

The surface of the pressure sensor during the limb immobilization phase is 1 cm^2^, according to 1 N = 1 Kg/s^2^ the pressure values read by the sensor will have the dimensions of $\mathrm{gf}/{\mathrm{cm}}^{2}.$ Figure 18 shows the detected mean pressure. As illustrated, during the normal state, the value of the pressure is constant and is equal to 60 gf/cm^2^. However, when a slight pressure is produced due to an inflammatory process, the sensors are able to perfectly detect an increment of this pressure. In order to simulate what occurs in an inflammatory process, a force over the splint was applied between minutes 7 and 10. This variation was detected by the sensors, reading a pressure that rose from 60 gf/cm^2^ to 100 gf/cm^2^. Therefore, when the pressure was increased due to the inflammation of the area, the sensors perfectly detected the increment of the mass inside the splint [16]. The graph shows the actual and historical pressure. Real-time monitoring and the collection of historical data on the pressure variation of the injured area during the immobilization period allows the specialist to control inflammatory phenomena.

### 3.3. Colour

**Figure 19:**
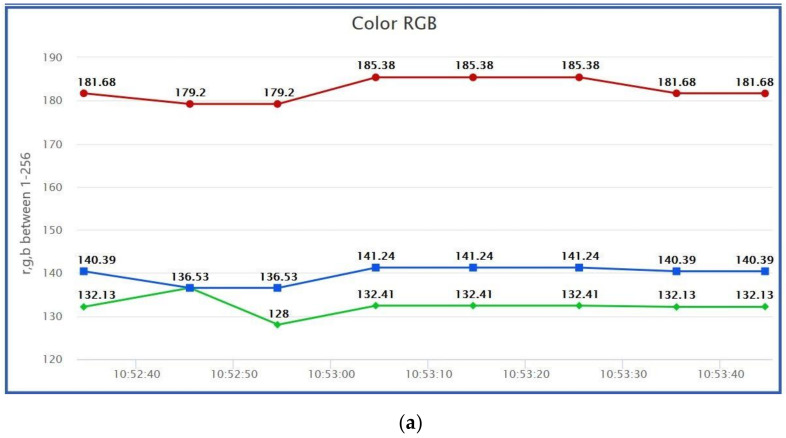

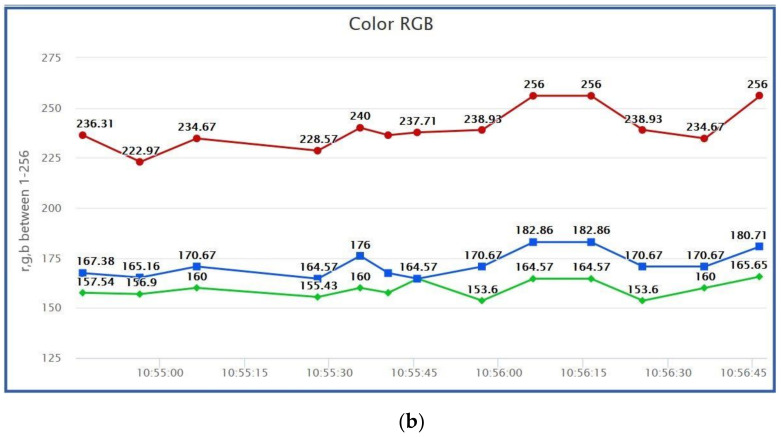
Color RGB sensor lectures without hematoma (**a**) and with hematoma (**b**).

The test was done on the skin of a volunteer that had a hematoma, and was compared to another part of his body, that had no signs of hematoma. The sampling of the test was carried out with a frequency of 10 s. It is possible to see in Figure 19 that the sensor is capable of detecting a color change in the skin. In Figure 19a, the skin has a normal color with no signs of hematoma, whilst in Figure 19b the sensor detects an increment in the RGB values due to the signs of hematoma. Due to this, it will be possible to check the evolution of the color during the very initial stages of the treatment, but also during posterior stages. It is possible to see that the three values are incremented, but it is specifically the 38% increment of the red value that makes it possible to detect the hematoma.

### 3.4. Diagnostic

The knowledge of these data makes it possible to identify temperature, humidity, pressure or skin color changes. Combining them, makes it possible to identify signs of inflammation and detect possible problems during the treatment.

To accomplish this, an algorithm that can be configured to consider temperature and pressure changes during a period of time was programmed, to consider these data to be produced by an inflammation process but not by a position of the limb over a specific object. The considered parameters are showed in Table 5. However, these parameters are showed as an example of the possibilities of the system, with no real medical considerations in this case. The timing would be the necessary to distinguish a value due to inflammation from a collision or resting state position. Moreover, to avoid a wrong measure of the pressure sensors, it may be considered to add a third sensor in opposite position. An inflammation process will produce an increment volume inside the splint, and an increment of the pressure in all the sensors, in a simultaneous and cotemporary way. About the range, it is proposed a 5%–10% change of the value due to the acquired data during the tests.

Moreover, the combination of this information with color and humidity that can also be consulted remotely by a specialist will result in a complete diagnosis.

## 4. Discussions

This study shows the combination of traditional immobilization techniques with new 3D modelling and prototyping techniques. This new system allows us to follow the evolution of the injured part in a very detailed way. Moreover, it is possible to apply treatments that improve not only the timing but also the quality of the rehabilitation. As previously explained, in this study electrostimulation, drainage, iontophoresis, laser and ultrasound have been considered as treatments. However, the potential of these kinds of 3D techniques makes it possible for other medical therapies to also be considered and included during the design process, according specialist dictated requirements.

These treatment possibilities are combined with the acquired data to even improve the treatment. Adding sensors to these splints, makes it possible to analyze in real time how the therapy is functioning and new steps to get the most progress.

Furthermore, the vast capacities that remote monitoring allows would simplify the revisions that must be carried out by a specialist. This allows doctors to follow the progress of more patients, by using objective data. These data may be collected into a database, in order to compare different evolutions of different treatments in similar injuries.

For this study, the use of ruggedized sensors was not considered, as the main purpose of the study is to show the new possibilities of using sensors in splints, and the benefits of using new technologies in this kind of treatment. The performance and resolution of the implemented sensors, were correct for the tests, as it is possible to see in the different figures. Humidity sensor DHT22 has an error due to hysteresis of +-0.3%. The sensor is looking for an increment of the internal humidity in comparison with the external humidity. The value will be around 3%–5% higher when a sweating process is produced. Due to this, the error is not significant and will not interfere in the correct detection of a sweating process. In this case, it is completely functional.

From a medical point of view, there is no necessity to know the absolute value, or trying to get an optimal value, as this will depend on the patient and lesion. This is why the main focus of the study is to analyze the new capabilities by detecting the variations on the acquired data.

The design of the different windows includes round edges to avoid any kind of chafing or similar. In no case the design of these windows may cause injury to the affected limb or by edge effect since the additive manufacturing design allows smoothing all the edges in contact with the patient, nor by suction effect on the skin [75] as the splint is not indicated for support in load nor produce its own vacuum because it is an open design. Furthermore, it is mandatory to use biocompatible materials [76,77] to avoid any kind of irritation, eschar or ulcer.

The design of the splint is made according to the affected anatomic area of each patient. The design includes an offset to suit perfectly over the area. The design and the position of the windows must be supervised by a medical specialist, according to the lesion. These windows allow a visual control of the treatment, what is especially critical in the initial stages. However, the windows do not intervene in the treatment. In cases where the use of splints indicated for support and gait of the lower limb is required, the corresponding structural study would be carried out, and accessible windows should be generated only at the time of use through the design of a closure. Moreover, this kind of module would allow us to take off the splint to clean the skin if needed, something not possible with traditional splints.

## 5. Conclusions

The main objective of this study is to show an evolution of traditional inmobilization methods. It is not possible to apply any kind of rehabilitation before removing the splint using a traditional inmobilization splint. Moreover, it is not possible to know what is happening under the splint.

This study shows the possibilities of using individualized immobilization splints by Advanced Manufacturing techniques scanning and prototyping. It is possible to fabricate a splint that fits perfectly on each limb. Moreover, it creates some areas for applying different treatments that produces at least a 30% decrease in the treatment period compared to plaster splints.

On the other hand, it is possible to incorporate some sensors for real time acquisition of data from inside the splint. This will allow health practitioners/doctors to anticipate treatment for different issues that may appear.

The electronic device that gets the data from the different sensors is able to combine and analyze the information received from the data. After that, it may send alerts when detecting inflammatory processes, skin color changes, or high humidity values. These alerts are shown on a display or a mobile device or may also be consulted remotely.

For future studies, the creation of a database where different smart splints send the acquired data is proposed as an improvement. This way, by using the new Big Data concept and Data Science, the device would be able to learn from the different symptoms to avoid problems before they happen and also to design specific treatments for each lesion and patient.

## Figures and Tables

**Table 1:** Technical specifications of the sensors.

Sensor Serial Number	DS18B20	DHT22	DF9-40	TCS34725
Dimensions (mm)	6 × 6 × 50	15 × 7.7 × 20	40 × 20 × 0.25	25 × 20 × 1.5
Power voltage (V)	3.0–5.5	3.3–6	5	5
Working range	−55 °C to 125 °C	−40 °C to 80 °C 0 to 100% RH	0–500 g	-
Resolution	±0.0625 °C	0.1 °C, 0.1% RH	14.5 g	-

**Table 2:** Technical specifications of Sense™ 3D scanner.

**Maximum Scan Volume**	2 × 2 × 2 [m]
**Minimum Scan Volume**	0.2 × 0.2 × 0.2 [m]
**Working Distance**	0.2–1.6 [m]
**Number of Cameras n**	2
**Class Certified Laser Product**	1
**Resolution at 0.5 m**	1 [mm]

**Table 3:** Mechanical properties [65].

Properties	Units	PLA
ρ (Polymer density)	g/cm^3^	1.21–1.25
σ (tensile strength)	MPa	21.0–60.0
E (tensile modulus)	GPa	0.35–3.50
ɛ (ultimate strain)	%	2.50–6.00
σ_s_ (specific tensile strength)	Nm/g	16.8–48.0
E_s_ (specific tensile modulus)	kNm/g	0.28–2.80
Tg (glass transition temperature)	°C	45–60
Tm (melting temperature)	°C	150–162

**Table 4:** Different parameters used for the manufacturing of the leg splint.

Layer height [mm]	0.2
Extruder [mm]	0.4
Density [%]	40
Thickness perimeter each layer [mm]	1
Print speed [mm/s]	60
Temperature [°C]	220

**Table 5:** Algorithm configuration to detect inflammation.

Temperature increment	1.5 °C
Pressure increment	60 gf/cm^2^
Time during temperature increment	60 m
Time during pressure increment	60 m
